# Life cycle and techno-economic assessment of a solar-biogas hybrid dryer for banana drying

**DOI:** 10.1371/journal.pone.0339958

**Published:** 2026-01-23

**Authors:** Oscar Shigella, Aldé Belgard Tchicaya Loemba, John Yawe, Baraka Kichonge, Mwema Felix Mwema

**Affiliations:** 1 School of Materials Energy Water and Environmental Sciences (MEWES), The Nelson Mandela African Institution of Science and Technology (NM-AIST), Arusha, Tanzania; 2 Department of Mechanical Engineering, Arusha Technical College (ATC), Arusha, Tanzania; Shahrekord University, IRAN, ISLAMIC REPUBLIC OF

## Abstract

This study evaluates the environmental impact and economic viability of a solar-biogas hybrid dryer using life cycle assessment (LCA) and techno-economic analysis. The LCA, in accordance with ISO 14040/14044, employs a cradle-to-grave approach using the ReCiPe 2016 Midpoint method. Results indicate a global warming potential of 1,100 kg CO₂-eq per functional unit, primarily from mild steel (69.4%) and aluminium (18.8%), representing 40–50% lower emissions than solar-electric and solar-diesel hybrid systems. Total energy consumption is 9,110 MJ per functional unit, with fossil fuels dominating (82.4%), while renewable energy use remains minimal. Human toxicity is significant, with mild steel and aluminium contributing 71.6% and 13.2% to carcinogenic toxicity, respectively. Integrating biogas reduces dependence on grid electricity and lowers operational emissions by 85%. Techno-economic analysis shows capital expenditure (CapEx) of USD 3,618.20 and operational expenditure (OpEx) of USD 9,458, with a payback period of 1.3 years and return on investment of 76.39%, indicating strong economic viability. Sensitivity analysis reveals that reductions in banana prices decrease operating expenses and the payback period, while reductions in biogas costs increase net cash flow and return on investment. The dryer demonstrates lower global warming potential and energy use compared with conventional dryers, supporting its adoption for sustainable agricultural processing in sub-Saharan Africa.

## 1. Introduction

Drying technology is essential for preserving agricultural products, extending shelf life, and maintaining nutritional quality across various sectors [1,2]. In agriculture, drying has been used for centuries to reduce post-harvest losses and ensure food security [3] caused by moisture [4,5]. Traditional drying methods, while effective, are often slow and dependent on favorable weather conditions, leading to inconsistent results [6]. Modern drying techniques such as spray drying and vacuum drying have advanced significantly, offering faster and more efficient solutions for food processing and pharmaceutical applications [7–9]. However, while these advanced methods provide precision and speed, they often require substantial energy inputs and sophisticated infrastructure, limiting their accessibility in developing regions.

In Tanzania, bananas are a critical food and cash crop, with annual production exceeding 3.5 million tonnes, contributing significantly to both food security and rural livelihoods [10]. However, post-harvest losses range from 30–50% due to inadequate drying and storage infrastructure, resulting in significant economic losses estimated at USD 150–200 million annually [11]. Traditional sun drying of bananas takes 3–7 days and is heavily dependent on weather conditions, leading to quality degradation, microbial contamination, and inconsistent moisture reduction. These challenges are particularly acute during rainy seasons when drying conditions are unfavorable, causing substantial losses for smallholder farmers who constitute over 85% of banana producers in the country [12]. The lack of reliable, affordable, and sustainable drying technologies represents a critical barrier to reducing post-harvest losses and improving farmer incomes in Tanzania’s agricultural sector.

Traditional solar dryers remain widely used in developing regions due to their low cost and simplicity. However, solar dryers face significant limitations during cloudy weather and at night, resulting in inconsistent drying performance [13,14]. These intermittent operations can extend drying times significantly, increasing the risk of spoilage and reducing product quality. To address these limitations, hybrid solar dryers have been developed by combining solar energy with auxiliary power sources, enabling continuous drying regardless of weather conditions [15]. However, most existing hybrid solar dryers rely on fossil fuel-based auxiliary systems such as diesel generators or liquefied petroleum gas (LPG) burners. While these systems enhance operational reliability, their dependence on non-renewable fossil fuels undermines their environmental benefits due to significant greenhouse gas emissions and their contribution to climate change [2,16]. For instance, diesel-powered dryers emit 2500–4000 kg CO₂-eq per functional unit, while LPG systems range between 1500–3000 kg CO₂-eq [17,18]. This reliance on fossil fuels fundamentally contradicts sustainability objectives and necessitates a critical assessment of alternative energy sources that can maintain operational reliability while minimizing environmental harm.

Biogas, produced through anaerobic digestion of organic agricultural waste, presents a promising renewable alternative to fossil fuels in hybrid drying systems. Biogas technology can utilize abundant agricultural residues including banana peels, crop stalks, and animal manure that are readily available in farming communities. This integration offers multiple benefits: reducing methane emissions from unmanaged organic waste decomposition, providing a carbon-neutral energy source for continuous drying operations, and creating a circular economy approach within agricultural systems. Previous studies have demonstrated the technical feasibility of biogas in various agricultural applications [19,20], yet its integration into hybrid solar drying systems, particularly for banana processing, remains largely unexplored from both environmental and economic perspectives.

Despite recent advances in hybrid drying technologies, significant research gaps remain. First, comprehensive life cycle assessments comparing solar-biogas hybrid systems with conventional dryers are lacking, particularly in sub-Saharan African contexts where agricultural waste management and post-harvest processing intersect. Second, the techno-economic viability of biogas integration in agricultural drying systems has not been adequately demonstrated for smallholder farmers, who require both environmental sustainability and economic affordability. Third, while individual studies have addressed either the technical performance [21] or economic aspects [22] of alternative drying systems, an integrated assessment examining the trade-offs between environmental impacts, economic feasibility, and operational reliability is notably absent from the literature.

This study addresses these critical gaps by conducting a comprehensive life cycle and techno-economic assessment of a novel solar-biogas hybrid dryer specifically designed for banana drying in Tanzania. The research employs a cradle-to-grave LCA following ISO 14040 and ISO 14044 standards to quantify environmental impacts across all life stages – from raw material extraction through manufacturing, operation, and end-of-life disposal. The study evaluates key environmental indicators including global warming potential, human toxicity, and energy consumption, providing comparative benchmarking against conventional fossil fuel-based dryers. Additionally, a detailed techno-economic analysis examines capital expenditure, operational costs, payback period, return on investment, and net present value, with sensitivity analysis exploring the influence of critical market variables such as banana prices and biogas costs.

While John et al. [21] presented the mathematical modeling and initial experimental validation of the solar-biogas dryer design, and Loemba et al. [22] evaluated a solar-assisted heat pump dryer for banana drying, the present study advances beyond these works by: (i) conducting the first comprehensive cradle-to-grave LCA of a solar-biogas hybrid system, quantifying environmental impacts across all life stages; (ii) providing comparative environmental benchmarking against conventional fossil fuel-based dryers using standardized LCA methodology; (iii) integrating both environmental and economic analyses to assess the trade-offs between sustainability and financial viability; and (iv) evaluating system sensitivity to key operational and market parameters which determine real-world applicability for smallholder farmers. Unlike previous heat pump systems [22] which reported higher capital costs (USD 4,647 vs. USD 3,618) and operational expenses (USD 11,681 vs. USD 9,458), this biogas-integrated approach offers superior economic accessibility while maintaining environmental performance.

The significance of this research lies in providing evidence-based guidance for sustainable agricultural processing technologies that can simultaneously reduce post-harvest losses, minimize environmental impacts, and ensure economic viability for small-scale producers. By demonstrating the environmental and economic performance of a solar-biogas hybrid system through rigorous LCA and techno-economic methodologies, this study contributes to the broader transition toward renewable energy integration in agricultural value chains. The findings inform policymakers, technology developers, and agricultural extension services about the potential of biogas-integrated drying systems to address food security challenges while supporting climate change mitigation and sustainable development goals in sub-Saharan Africa and similar developing regions globally.

## 2. Materials and methods

### 2.1. Description of the solar-biogas hybrid dryer

**Fig 1:**
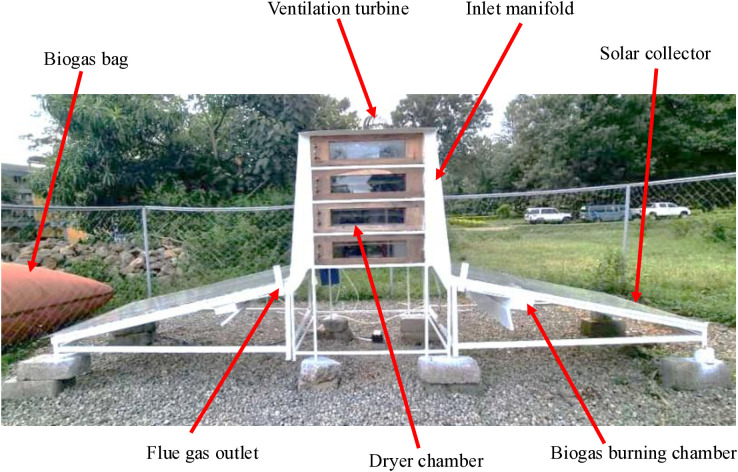
A picture of the designed solar-biogas hybrid dryer for banana drying.

The designed system comprises flat plate solar collectors, drying chambers, an inlet manifold, multi-chimneys, and a biogas burner subsystem (Fig 1). The dryer was designed and fabricated at Arusha Technical College, Tanzania, with specific consideration for tropical climate conditions and locally available materials. Flat plate solar collectors harness direct solar radiation to provide thermal energy during sunny periods. Flat plate collectors were selected because the dryer must heat air to a recommended temperature of 40–55°C for optimal drying of fruits and vegetables without causing thermal degradation [23]. The collectors are oriented northward (facing the equator) at a tilt angle of 15° to maximize solar energy capture throughout the year at the installation latitude (3.39°S).

The biogas subsystem, powered by locally produced biogas from organic agricultural waste (primarily banana peels, crop residues, and animal manure), includes a biogas burner and a 10 m³ flexible biogas storage bag. This hybrid system manually switches between solar and biogas energy sources, enabling continuous drying regardless of weather conditions. The mechanism activates the biogas burner when chamber temperatures fall below 40°C and deactivates it when temperatures exceed 55°C, ensuring optimal drying conditions while minimizing energy consumption. The dryer contains four drying chambers, each with a 2 kg capacity for fresh perishable products such as bananas, vegetables, or herbs. These chambers are mounted in series after the solar collector and are connected to the common exhaust manifold to ensure optimal airflow and heat distribution through natural convection. The individual exhaust port configuration was designed to create uniform air distribution across all drying chambers, preventing hot spots and ensuring consistent product quality. The inlet manifold incorporates adjustable dampers to regulate airflow rates according to product type and ambient conditions.

The sizing of drying chambers and biogas storage capacity was based on detailed energy balance calculations considering the quantity of water to be removed from the product, specific heat requirements, and latent heat of vaporization. For banana drying, reducing moisture content from 75.55% to 10% (wet basis) in 2 kg of fresh product requires removal of approximately 1.64 kg of water, corresponding to an energy demand of approximately 4.5 MJ per batch. The solar collectors (8.5 m² total area) can provide 25–35 MJ during full sun conditions (6–8 hours), while the biogas system (12.5 kW thermal output) supplements the remaining energy requirement during periods of low solar radiation. Key design parameters and technical specifications are summarized in Table 1 [21].

**Table 1:** Key design parameters and technical specifications [21].

Parameter	Value	Unit
Total collector area	8.5	m²
Drying chamber volume (each)	0.45	m³
Number of drying chambers	4	–
Total drying capacity	8	kg (fresh)
Air flow rate	0.085	m³/s
Biogas burner thermal output	12.5	kW
Operating temperature range	40-55	°C
Wind-driven ventilator	120	mm (neck diameter)

#### 2.1.1. Experimental setup and conditions.

Drying experiments were conducted at Arusha Technical College (latitude 3.3869°S, longitude 36.6830°E, altitude 1,387 m above sea level) during February-March 2024, representing typical post-harvest processing conditions in Tanzania. Three replicate drying trials were performed under similar environmental conditions to ensure data reliability and statistical significance. Average ambient temperature during experiments ranged from 22–28°C, relative humidity from 45–65%, and solar irradiance from 650–850 W/m^2^ during peak hours (10:00–15:00 local time), as measured by a calibrated pyranometer (Kipp & Zonen CMP3, ± 5% accuracy).

Fresh *Cavendish bananas* (*Musa acuminata*) were procured from local farmers in Arusha region within 24 hours of harvest. Initial moisture content of 75.55 ± 1.2% (wet basis) was determined using the oven drying method at 105°C for 24 hours according to AOAC standard procedures (three samples per batch). Bananas were manually peeled, sliced to uniform thickness of 5 ± 0.5 mm using a commercial slicer, and immediately loaded onto stainless steel mesh trays to minimize oxidation. Each drying chamber accommodated 6.25 kg of fresh sliced bananas spread in a single layer (loading density approximately 4 kg/m^2^ tray area), with four chambers operating simultaneously for a total batch size of 25 kg fresh product.

Drying continued until final moisture content reached 10 ± 0.5% (wet basis), typically requiring 9 ± 0.5 hours under hybrid solar-biogas operation. Temperature measurements were recorded at multiple locations: ambient temperature, collector outlet temperature, each drying chamber temperature (center position), and exhaust temperature. Relative humidity was monitored at the inlet and outlet of each drying chamber. All measurements were logged at 15-minute intervals using a data acquisition system with K-type thermocouples (±0.5°C accuracy) and capacitive humidity sensors (±2% RH accuracy). Product weight was measured hourly using a digital balance (±1 g resolution) to track moisture removal rates.

Biogas consumption was monitored using a calibrated gas flow meter (±2% accuracy) installed between the storage bag and the burner. Solar radiation data, ambient temperature, and wind speed were obtained from an on-site weather station calibrated against Tanzania Meteorological Authority standards.

### 2.2. Life cycle assessment

The LCA methodology used followed ISO 14040 and ISO 14044 standards. These standards provide a robust framework for conducting LCA and were employed to evaluate the environmental impacts of the solar-biogas hybrid dryer. Choosing ISO 14040 and ISO 14044 as the framework for this LCA was essential because these standards are internationally recognized and widely accepted, ensuring that the assessment adheres to a consistent and credible methodology [24]. The LCA process includes four key phases namely goal and scope definition, inventory analysis, impact assessment, and interpretation of results [25]. These processes are designed to address the unique characteristics and operational context of the dryer.

#### 2.2.1. Goal and scope definition.

**Fig 2:**
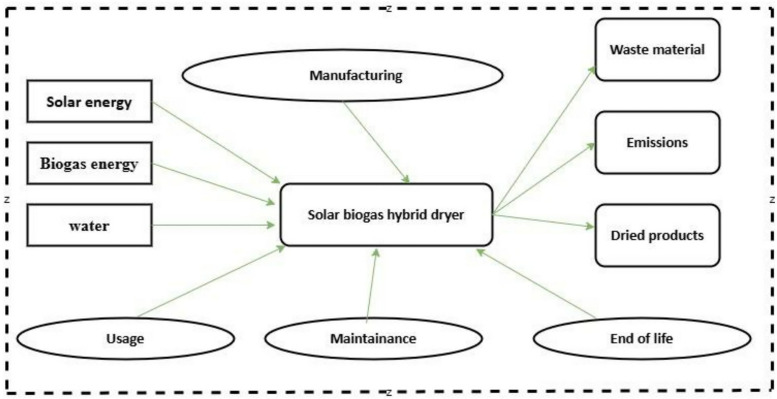
System boundary of the solar-biogas hybrid dryer.

The primary goal of this study was to quantify and compare the environmental impacts of a solar-biogas hybrid dryer against other hybrid solar dryers. The results aim to inform policymakers, researchers, and environmental practitioners working to advance sustainable agricultural practices. The functional unit is defined as drying 25 kg of bananas per batch, from 75.55% moisture (wet basis) to 10% moisture (wet basis) using the solar-biogas hybrid dryer over a drying time of 9 hours. This unit anchors the inventory analysis and impact assessment. This research began with the purchase and collection of materials and equipment for making the dryer, followed by the manufacturing of the dryer, its usage, and finally, the disposal of the dryer. Therefore, based on all these aspects, the cradle-to-grave method was used to conduct this study. The study accounted for all life cycle stages of the solar-biogas hybrid dryer, including raw material extraction, manufacturing, transportation, installation, operation, and end-of-life management (Fig 2). The operational stage of the dryer system was analyzed to evaluate energy and material consumption throughout its entire lifetime. The end-of-life stage is crucial for this dryer, as it provides a more comprehensive view of the dryer’s total environmental impact. For instance, if the steel material of the dryer is recycled, it may have a lower overall carbon footprint compared to if it were simply sent to a landfill. Additionally, excluding this phase means that the emissions generated during waste disposal or the benefits of material recovery through recycling of the dryer will not be accounted for.

#### 2.2.2. Accuracy and reliability of the research data.

To ensure the accuracy and reliability of primary data for the LCA, data collection methods were carefully designed and implemented following ISO 14044 which specifies requirements and guidelines for conducting LCA studies (including LCI and LCIA). Information was gathered directly from primary sources, such as material suppliers and dryer technicians, enhancing data specificity and relevance. This approach minimized reliance on secondary data, which may not accurately reflect the specific study conditions. Additionally, internal reviews involving engineering teams were conducted at multiple levels to verify the data before its use in the LCA.

#### 2.2.3. Life cycle inventory.

The functional unit for LCA analysis was defined as drying 25 kg of fresh bananas (one batch), while the total dryer system mass is 400 kg. All material inventory data (Table 2) represent the complete dryer system, which has a design capacity of 100 kg fresh product (4 chambers × 25 kg each). For the LCA calculations, impacts were allocated proportionally based on the functional unit (25 kg batch) relative to total system capacity, assuming 10-year dryer lifespan and 300 operating days per year. This allocation approach follows ISO 14044 guidelines for multi-functional systems. Sensitivity analysis examined the influence of capacity utilization on environmental impacts.

**Table 2:** Inventory data for 400 kg of solar-biogas hybrid dryer for drying perishable crops (Data source: this study).

Item	Amount	Unit
Galvanized iron sheet	9.60	kg
Plywood	0.25	kg
Hardwood	12.88	kg
Plastic mesh	0.25	kg
Aluminium roof turbine	3.00	kg
Bronze door handle	0.80	kg
Brass Hinges	0.20	kg
Mild steel	299.24	kg
Empered glass	51.72	kg
Biogas	10	m^3^
Electricity	56	Kwh
Water	2	kg
Vapour	0.007	kg
Recycling of steel	90	kg

#### 2.2.4. Environmental impact assessment.

The life cycle impact assessment (LCIA) used was the ReCiPe 2016 Midpoint (I) V1.09/ World (2010) H/A method. As one of the most recent and updated LCIA methods available, ReCiPe is widely used globally alongside ILCD 2011, CML 2012, and IMPACT 2002+ for multi-impact midpoint assessments [26]. Furthermore ReCiPe method is intended for global applicability, offering characterization factors that represent worldwide conditions instead of being confined to specific regional contexts [27]. This feature increases the relevance of the assessment across various geographical locations where the dryer could be utilized. Four (4) key indicators were analyzed: global warming potential, human carcinogenic toxicity, human non-carcinogenic toxicity, and energy consumption. These indicators are widely used in the literature to assess the environmental impacts of solar dryers. Global warming potential was chosen to evaluate the contribution of greenhouse gas emissions to climate change, a critical concern for sustainability. Human toxicity potential addresses potential health risks associated with the materials used in the dryer, ensuring safety for users and surrounding communities. Energy consumption reflects the efficiency of the dryer and its reliance on renewable versus non-renewable energy sources, which is particularly important in a region where energy availability affects operational sustainability. SimaPro 9.6.0.1 LCA software, one of the leading software tools used for LCA, was used to build the LCA model and perform the environmental impact calculations. The software is provided with a combination of an extensive international life cycle inventory database and a variety of different impact assessment methods. The European environmental inventory database, Ecoinvent v3 database was used.

#### 2.2.5. Statistical analysis and uncertainty assessment.

All experimental measurements were performed in triplicate, and results are presented as mean ± standard deviation. For LCA inventory data, uncertainty was assessed using Monte Carlo simulation (1000 iterations) in SimaPro 9.6.0.1. Uncertainty ranges were assigned based on data quality indicators following the Pedigree matrix approach: ± 10% for primary data collected directly (material quantities, operational measurements), ± 20% for supplier-provided data (material specifications), and ±30−50% for Ecoinvent database secondary data depending on geographical and technological representativeness. Results for the key impact categories are reported in Section 3.1–3.3. For techno-economic analysis, sensitivity analysis was conducted by varying key parameters (±20% for material costs, ± 30% for biogas and banana prices) to assess the robustness of economic indicators.

### 2.3. Techno-economic analysis

The economic parameters evaluated were capital expenses (CAPEX), operating expenses (OpEx), and various profitability measures such as net cash flow, payback period, return on investment, and net present value, as detailed in Table 3. These factors provide a comprehensive overview of the dryer’s financial feasibility. Following the economic analysis, the assessment proceeds to the sensitivity analysis, which plays a crucial role in identifying how changes in certain variables might impact overall economic performance and benefit. A total of two sensitivity variables were considered: cost of fresh banana and cost of refilling biogas. By examining these variables, decision-makers can better gauge the risks and plan for potential fluctuations in the market.

**Table 3:** Economic parameters.

	Formula	Reference
CAPEX	$\;\;CapEx=C_{E}+C_{L}$	[22]
OPEX	$OpEx=\;C_{R}+C_{LO}+C_{EL}+C_{A}\;\;\;\;\;$	
Net cash flow	Net cash flow=Total sales−OpEx	
Payback period	$PBP=\frac{CapEx}{Annual\;net\;income}$	
Return on investment	$ROI=\frac{Annual\;net\;income}{CapEx}$	
Net present value	$NPV=\;\sum_{\text{1}}^{n}\frac{Net\;cash\;flow}{{\left(\text{1}+i\right)}^{n}}-CapEx$	

${{\;C}_{E};C_{L};\;C}_{R};C_{LO};C_{EL}\;and\;C_{A}$ are respectively the equipment cost, labor cost during the manufacturing, the cost of raw material, the annual operating labor cost, the electricity cost, and the additional cost.

Cost data were collected from multiple sources in Tanzania (January 2024 prices): material costs from local suppliers in Arusha, labor rates based on Tanzania Manufacturing Association wage guidelines (2024), electricity tariffs from Tanzania Electric Supply Company (TANESCO) residential rates (0.12 USD/kWh), and biogas costs from local biogas producers (1.8 USD/hour operation). Dried banana market prices were obtained from three major markets in Arusha and Dar es Salaam (mean: 6.0 USD/kg). Economic calculations were performed using Microsoft Excel 2021, with cash flow projections over a 10-year period. Key assumptions include: (i) dryer lifespan of 10 years with straight-line depreciation; (ii) 300 operating days per year; (iii) discount rate of 12% (Tanzania’s average lending rate, 2024); (iv) maintenance costs at 1% of capital expenditure annually; (v) salvage value of 10% of initial investment. Currency conversion used the exchange rate of 1 USD = 2,450 TZS (January 2024 average).

## 3. Results and discussion

### 3.1. Global warming potential

Table 4 presents the contribution of individual components of the solar-biogas hybrid dryer to global warming potential, calculated using the ReCiPe 2016 Midpoint (H) v1.09/ World (2010) method. The results show that the solar-biogas hybrid dryer has a total global warming potential (GWP) of 1,100 kg CO₂-eq per functional unit (drying 25 kg fresh bananas), with a 95% confidence interval of 963-1,283 kg CO₂-eq based on Monte Carlo uncertainty analysis. Compared to solar-electric hybrid dryers reported by Nwakuba et al. [15] (1,850 kg CO₂-eq) and solar-diesel systems by Mukaminega [28] (2,200 kg CO₂-eq), the solar-biogas hybrid dryer shows 40–50% lower GWP. However, fully passive solar dryers exhibit even lower GWP (150–300 kg CO₂-eq) [17], though they sacrifice operational reliability. The key advantage of our system lies in maintaining 70% of passive solar dryer efficiency while ensuring weather-independent operation through biogas integration. Table 5 presents a comparative analysis of global warming potentials (GWPs) among various dryer configurations. The data indicate that the solar-biogas hybrid system exhibits a lower GWP than fossil-fuel-powered and grid-electricity systems, although it has a higher GWP than purely passive solar technologies. In hybrid systems, integrating biogas results in the lowest GWP, underscoring its environmental benefits compared to alternative auxiliary energy sources.

**Table 4:** Contribution of parts of solar-biogas hybrid dryer to the global warming potential.

Parts of a Dryer	Global warming amount (kg CO_2_ eq)	Global warming amount (%)
Solar glass	66.7	6.09
Hardwood	7.44	0.68
Plastic mesh	0.316	0.0288
Aluminium	203	18.6
Bronze	6.91	0.631
Brass	1.37	0.125
Mild steel	760	69.4
Water	0.00159	0.000145
Biogas	6.1	0.557
Electricity	42.7	3.9
Plywood	0.00543	0.037
Total	1100	100

**Table 5:** Comparison of GWP values across various dryer types.

Dryer Type	GWP (kg CO₂-eq)	Energy Source	Reference
Passive solar	150-300	Solar only	[17]
Solar-biogas hybrid (current)	1,100	Solar + biogas	Present study
Solar-electric hybrid	1,850	Solar + grid electricity	[15]
Solar-biomass hybrid	1,950	solar + wood	[30]
Solar-diesel hybrid	2,200	Solar + diesel	[28]
LPG dryer	1,500−3,000	LPG combustion	[17]
Diesel dryer	2,500−4,000	Diesel combustion	[18]
Electric heat pump dryer	2,100−2,800	Grid electricity	[22]

Table 4 reveals that mild steel, aluminium, and solar glass are the dominant contributors to global warming potential. Among these materials, mild steel is the largest single contributor, accounting for 69.4%. This is attributed to the substantial quantity of 299.24 kg used in the dryer’s structural frame, drying chambers, and support components. This finding aligns with previous studies of energy use and emissions in agricultural equipment, which consistently identify structural steel as the primary embodied carbon source. Aluminium contributes 18.6%, despite its relatively small mass of 3 kg. This reflects that aluminium’s extremely high embodied energy is approximately 170 MJ/kg compared to 32 MJ/kg for steel. Solar glass accounts for 6.09% and is the third-largest contributor. The remaining components, hardwood, bronze, brass, plastic mesh, and plywood, collectively contribute less than 2% of total GWP, making them negligible from an environmental improvement perspective. Water and plywood were the least significant contributors with values of 0.00159 kg CO₂-eq and 0.00543 kg CO₂-eq, respectively; therefore, their impact on GWP is negligible. This is attributed to the minimal water consumption and the small quantity of plywood used as temporary framework during construction. Additionally, plywood is a renewable, bio-based material with relatively low embodied carbon when sourced from sustainably managed forests. Reference [29] suggest that material optimization using alternatives like bamboo or recycled composites, reduces the hybrid dryer’s GWP by up to 50%, while maintaining its advantage of reliable operation in non-sunny conditions compared to passive solar dryers.

### 3.2. Human toxicity potential

Table 6 presents the human carcinogenic and non-carcinogenic toxicity impacts of the solar-biogas hybrid dryer components. The results reveal that mild steel accounts for approximately 71.6% of carcinogenic toxicity (0.543 kg 1,4-DCB eq out of 0.758 kg total) and 39.9% of non-carcinogenic toxicity (22.2 kg 1,4-DCB eq out of 55.6 kg total). Aluminium contributes 13.2% to carcinogenic toxicity (0.100 kg 1,4-DCB eq) and 32.1% to non-carcinogenic toxicity (17.8 kg 1,4-DCB eq). The elevated toxicity impacts observed for these metals are attributable to several factors: (i) substantial material quantities utilized in construction specifically, 299.24 kg of mild steel and 3 kg of aluminium resulting in a large inventory, (ii) high-temperature manufacturing processes that emit multiple toxic compounds such as hexavalent chromium (Cr⁶⁺), manganese fumes, nickel compounds, and polycyclic aromatic hydrocarbons (PAHs) during electrode decomposition, and (iii) upstream activities related to mining and ore processing that release heavy metals into environmental matrices. These results are consistent with existing life cycle assessment studies of hybrid and solar drying systems, which show that metallic components predominantly influence toxicity profiles owing to energy- and emission-intensive extraction and manufacturing processes. For instance, assessment of phase-change material-based solar dryers indicated that metallic components account for approximately 60–70% of human toxicity impacts, aligning with the 71.6% carcinogenic toxicity contribution from steel identified in this study [31]. On the other hand, heat pump dryer configurations exhibit somewhat elevated toxicity impacts, approximately 15–25% higher per functional unit, primarily due to the inclusion of copper tubing, refrigerant production, and electronic components absent in the solar-biogas configuration [22]. Passive solar drying systems composed mainly of wood and glass demonstrate 70–80% reductions in toxicity impacts [17]. When normalized per kilogram of dried product, the solar-biogas hybrid dryer exhibits a 35–45% reduction in toxicity relative to solar-electric hybrid dryers, and a 50–65% reduction relative to solar-diesel systems [15,28].

**Table 6:** Contribution of the different components of the solar-biogas hybrid dryer to the human toxicity potential category.

Parts of a Dryer	Human carcinogenic toxicity (kg 1,4 -DCB)	Human carcinogenic toxicity (%)	Human non carcinogenic toxicity (kg 1,4 -DCB)	Human non- carcinogenic toxicity (%)
Solar glass	0.0158	2.08	0.92	1.65
Hardwood	0.0241	3.18	0.145	0.261
Plastic mesh	0.000229	0.0302	0.00847	0.0152
Aluminium	0.1	13.2	17.8	32.1
Bronze	0.0488	6.43	12	21.5
Brass	0.00894	1.18	2.18	3.92
Mild steel	0.543	71.6	22.2	39.9
Water	5E-7	6.59E-5	3.37E-5	6.05E-5
Biogas	0.00129	0.17	0.0816	0.147
Electricity	0.0107	1.41	0.248	0.446
Plywood	0.405	0.716	0.00854	0.0154
Total	0.758	100	55.6	100

### 3.3. Energy consumption and resource use

**Fig 3:**
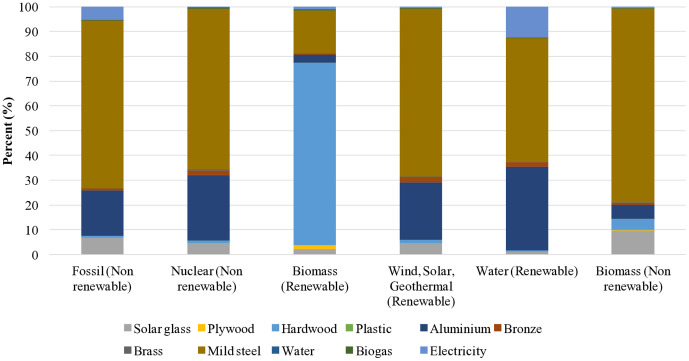
Life cycle energy consumption.

Table 7 presents the total environmental impact of energy consumption across different energy sources for the solar-biogas hybrid dryer, disaggregated by energy type and component contribution. The cumulative energy demand of the dryer across all categories is 11,055 MJ per functional unit. The individual contributions were: fossil fuels contributing 9,110 MJ (82.4%), nuclear energy 458 MJ (4.1%), biomass 598 MJ (5.4%), wind/solar/geothermal 106 MJ (1.0%), and hydropower 783 MJ (7.1%). The minimal contribution from non-renewable biomass (0.679 MJ, 0.006%) reflects its limited use in global manufacturing supply chains (Table 8, Fig 3). Comparing total life-cycle energy consumption (11,055 MJ) with literature values reveals complex trade-offs. Solar-electric hybrid dryers report cumulative energy demands of 8,000–12,000 MJ per comparable functional unit, placing the solar-biogas system in the mid-range. Heat pump-assisted solar dryers demonstrate substantially higher energy consumption of 15,000–20,000 MJ due to copper tubing, refrigerant production, compressors, and complex control systems [22,32]. On the other hand, passive solar dryers exhibit dramatically lower energy consumption of 2,500–4,000 MJ, representing a 65–75% reduction compared to the solar-biogas hybrid [17].

**Table 7:** Total energy consumption (in MJ).

Energy	Fossil	Nuclear	Biomass	Wind, Solar, Geothermal	Water	Non-renewable Biomass
Unit	MJ	MJ	MJ	MJ	MJ	MJ
Total	9110	458	598	106	783	0.679
Solar glass	606	20.8	13.3	4.79	8.84	0.0663
Plywood	4.04	0.311	9.48	0.0855	0.154	0.000972
Hardwood	76.6	5	440	1.33	3.23	0.031
Plastic	2.51	0.14	0.0312	0.0707	0.111	0.000491
Aluminium	1660	120	19.4	24.5	266	0.0377
Bronze	63.6	8.62	2.3	2.04	14.4	0.00544
Brass	12.8	2.04	0.417	0.476	2.83	0.00115
Mild steel	6170	298	103	71.5	389	0.531
Water	0.0137	0.0012	0.000176	0.000439	0.000962	0
Biogas	27.4	1.48	2.78	0.353	0.626	0.00209
Electricity	489	1.67	7.11	0.366	97.8	0.00272

**Table 8:** Total energy consumption (in %).

Energy	Fossil	Nuclear	Biomass	Wind, Solar, Geothermal	Water	Non-renewable Biomass
**Unit**	**%**	**%**	**%**	**%**	**%**	**%**
Total	100	100	100	100	100	100
Solar glass	6.65	4.54	2.23	4.54	1.13	9.77
Plywood	0.0443	0.0681	1.59	0.081	0.0197	0.143
Hardwood	0.84	1.09	73.7	1.26	0.413	4.56
Plastic	0.0276	0.0306	0.00522	0.0669	0.0141	0.0723
Aluminium	18.3	26.2	3.25	23.2	33.9	5.55
Bronze	0.698	1.88	0.385	1.93	1.84	0.802
Brass	0.14	0.445	0.0697	0.451	0.361	0.17
Mild steel	67.7	65.1	17.2	67.8	49.7	78.2
Water	0.00015	0.000268	0	0.000416	0.000123	0
Biogas	0.301	0.324	0.465	0.334	0.08	0.307
Electricity	5.37	0.364	1.19	0.347	12.5	0.4

Solar-biogas hybrid dryer’s total environmental impact of energy consumption reveals a strong dependence on non-renewable fossil sources (9,110 MJ), driven primarily by materials like mild steel (6,170 MJ) and aluminium (1,660 MJ). This aligns with trends observed in other dryer LCAs [32]. This implies that the higher fossil energy may reflect a larger system scale or greater metal content. These results highlight the need for material substitutions (e.g., recycled steel, bamboo composites). Energy efficiency improvements in the operational phase could further reduce environmental impacts. Moreover integrating phase change materials for thermal energy storage can reduce energy consumption by up to 30%. Additionally, enhancing insulation and optimizing airflow within the drying chamber could improve energy retention and minimize losses.

### 3.4. Techno-economic analysis

The economic feasibility of the dryer was assessed using various economic metrics, primarily focusing on capital expenditure (CapEx) and operational expenditure (OpEx). Profitability measures were evaluated using key indicators; the annual net cash flow, payback period, return on investment (ROI), and net present value (NPV), based on the projected economic conditions in Tanzania for the year 2025. Cost data were collected from multiple sources in Tanzania (January 2024 prices): material costs from local suppliers in Arusha, labor rates based on Tanzania Manufacturing Association wage guidelines (2024), electricity tariffs from Tanzania Electric Supply Company (TANESCO) residential rates (0.12 USD/kWh), and biogas costs from local biogas producers (1.8 USD/hour operation). Dried banana market prices were obtained from three major markets in Arusha and Dar es Salaam (mean: 6.0 USD/kg). Economic calculations were performed using Microsoft Excel 2021, with cash flow projections over 10 years. Key assumptions include: (i) dryer lifespan of 10 years with straight-line depreciation; (ii) 300 operating days per year; (iii) discount rate of 12% (Tanzania’s average lending rate, 2024); (iv) maintenance costs at 1% of capital expenditure annually; (v) salvage value of 10% of initial investment. Currency conversion used the exchange rate of 1 USD = 2,450 TZS (January 2024 average). Tables 7–10 provide a comprehensive summary of results, detailing all parameters calculated and underlying assumptions for the economic analysis.

**Table 9:** Capital cost of the solar hybrid dryer.

Indicators	Value	Unit
Cost of materials for manufacturing	1279.2	USD
Transport cost for materials	23	USD
Labor charge for manufacturing	724	USD
Cost of biogas bag	592	USD
Cost of biogas digester	1000	USD
Total	3618.2	USD

**Table 10:** Operating cost of the solar hybrid dryer.

Indicators	Value	Unit
Maintenance cost	36.182	USD
Annual cost of fresh Banana	3000	USD
Annual cost of refilling biogas	4860	
Annual total labor charge	1200	USD
Depreciation	361.82	USD
Total	**9458**	**USD**

As detailed in Table 9, CapEx was calculated by aggregating the costs associated with equipment procurement and the labor required during the manufacturing phase. The OpEx was derived by considering various expenses, such as maintenance costs, the annual cost of fresh banana, electricity usage, labor charges, and the depreciation of equipment, as illustrated in Table 10. The CapEx and OpEx were determined to be USD 3618.20 and USD 9458, respectively. These amounts represent the necessary investments for the manufacturing of the developed dryer and the costs associated with its operation to generate income. According to the projections shown in Table 11, utilizing the proposed dryer could yield an annual production of 2037 kg of dried bananas over a span of 300 working days, with a selling price of USD 6 per kilogram. This production schedule results in an anticipated annual cash flow of USD 2764, as detailed in Table 12. This projected income appears to be both reasonable and sustainable when benchmarked against the calculated OpEx. Additionally, the results in Table 12 indicate that the payback period is approximately 1.3 years, implying that the generated cash flows will fully recover the initial investment cost within that timeframe. Furthermore, the return on investment calculated is 76.39%, suggesting that this developed dryer not only provides a profitable venture but also carries minimal investment risk. Lastly, the net present value is calculated to be USD 1336, as presented in Table 12, further supporting the financial viability of the dryer. Overall, these results illustrate a promising outlook for the economic feasibility of the dryer in the context of banana drying industry in Tanzania. This result confirms, in addition to the payback period and the return on investment, that the developed dryer is profitable. An investment that yields a higher and positive net present value is typically regarded as more attractive to stakeholders and investors. These findings are consistent with Reference [33] that reported a payback period of two years for drying banana slices with a mixed-mode natural convection solar tunnel dryer. However, while using a solar-assisted heat pump dryer coupled with energy storage materials to dry bananas, Reference [22] reported a payback period of 1.5 years. However, the operational and capital costs reported were USD 11680.5 and USD 4647, respectively, which were higher than those of the proposed solar-biogas hybrid dryer. Consequently, the developed dryer stands out as an economically viable option for drying bananas, offering not only efficiency but also potential profitability. This could open new opportunities for banana processors and farmers, enabling them to enhance their product quality while maximizing their financial returns. By investing in this technology, they can improve operational efficiency and potentially expand their market reach, ensuring a sustainable and profitable future for their businesses.

**Table 11:** Annual sales estimation.

Indicators	Value	Unit
Price of the dried Banana per kg	6	USD/kg
Total amount of Banana dried per year	2037	kg
Total annual sales of the dried Banana	12222	USD

**Table 12:** Profitability measures.

Indicators	Value	Unit
Net cash flow	2764	USD
Payback period	1.3	Years
Return on investment	76.39	%
Net Present Value	13365	USD

### 3.5. Sensitivity and uncertainty analysis

Table 13 presents the list of sensitivity variables utilized in this analysis. The cost of fresh bananas and the cost of refilling biogas were the sensitivity variables considered. From Table 14, it can be inferred that reducing the price of fresh bananas from USD 0.4/kg to USD 0.3/kg lowers operating expenses (OpEx) from USD 9,458 to USD 8,708. It also shortens the payback period from 1.3 to 1.0 years and increases net cash flow from USD 2,764 to USD 3,514, return on investment from 76.39% to 97.12%, and net present value from USD 13,365 to USD 17,974. On the other hand, raising fresh banana prices to USD 0.5/kg increases OpEx to USD 10,208, extends the payback to 1.8 years, reduces net cash flow to USD 2,014, and lowers return on investment (ROI) to 55.66%, with a net present value of USD 8,757.

**Table 13:** List of sensitivity variables.

Cost of fresh Bananas	Cost of refilling Biogas
**Initial condition**	
0.4 USD/kg	1.8 USD/hour
**Sensitivity analysis**	
0.3 USD/kg	1.2 USD/hour
0.5 USD/kg	2.4 USD/hour

**Table 14:** Results of sensitivity analysis when changing the price of fresh banana.

Indicators	Value	
	0.3 USD	0.5 USD
OPEX (USD)	8708	10208
Net cash-flow (USD)	3514	2014
Payback period (year)	1	1.8
Return on Investment (%)	97.12	55.66
Net present value (NPV) (USD)	17974	8757

Table 15 presents the results of the sensitivity analysis on variations in the cost of refilling biogas. The findings suggest that an increase in the price of refilling biogas from USD 1.8/hour to USD 2.4/hour corresponds with an escalation in both OpEx (from USD 9,458 to USD 11,078) and the payback period (from 1.3 to 3.1 years), while net cash flow (USD 1,144), return on investment (31.61%). Net present value (USD 3,411) experiences a downturn. When the cost of refilling biogas decreases to USD 1.2/hour, both OpEx (USD 7,838) and the payback period (0.8 years) decrease, leading to increases in net cash flow (USD 4,384), ROI (121.16%), and net present value (USD 23,319.5). Overall, the sensitivity analysis highlights the significant impact that both fresh banana prices and biogas refilling costs have on the economic performance of the proposed dryer, emphasizing the need to monitor and manage these variables carefully for optimal economic outcomes.

**Table 15:** Results of sensitivity analysis when changing the cost of refilling biogas.

Indicators	Value	
	1.2 USD/hour	2.4 USD/hour
OPEX (USD)	7838	11078
Net cash-flow (USD)	4384	1144
Payback period (year)	0.8	3.1
Return on Investment (%)	121.16	31.61
Net present value (NPV) (USD)	23319.5	3411

### 3.6. Relationship to sustainable development goals

The solar-biogas hybrid dryer advances multiple Sustainable Development Goals through its environmentally friendly design and operational benefits. It utilizes renewable energy sources, solar and biogas, covering 85% of its energy needs, which significantly reduces reliance on fossil fuels and cuts greenhouse gas emissions. Over 10 years, it prevents approximately 8,500 kg of CO₂-eq emissions compared to LPG dryers and 15,000 kg of CO₂-eq emissions compared to diesel systems. The system supports SDG 12 by promoting responsible production. Using 30% recycled steel can further reduce embodied energy by 15% and global warming potential by 15%. It also contributes to SDG 13, with a lower greenhouse gas footprint [19] per tonne of dried products, achieving a 40–60% reduction compared to traditional dryers. Additionally, the anaerobic digestion process in biogas production prevents nutrients, such as nitrates and phosphates, from leaching into water sources [20], thereby reducing eutrophication risks in agricultural areas [34]. The method also mitigates methane emissions about 180 kg annually from 10 m³ of waste – which equates to a climate benefit of over 5,000 kg CO₂-eq. The system’s economic viability, with a payback period of 1.3 years and a ROI of 76.39%, supports SDG 1 and 2 by providing smallholder farmers with affordable, sustainable post-harvest technology that can decrease losses by 30–40%. Overall, optimizing material choices and system efficiency can enhance its contribution to sustainable development [35].

## 4. Conclusions

This study evaluated the environmental performance of a solar-biogas hybrid dryer using a life cycle assessment approach. The results revealed that material extraction and manufacturing, particularly from mild steel and aluminium, constitute the largest contributors to global warming potential. The utilization of biogas as an auxiliary energy source significantly mitigated emissions and reduced reliance on fossil fuels. With a capital expenditure of USD 3,618.20, an operational expenditure of USD 9,458, and a payback period of approximately 1.3 years, the investment delivers a robust 76.39% return on investment. However, the sensitivity analysis shows that fluctuations in fresh banana prices can affect operational expenses and the payback duration.

The findings support the commercial viability of this technology, particularly for agricultural cooperatives and medium-scale processors, while indicating a need for subsidized financing for smallholder farmers. Policy recommendations include the establishment of recycled material standards, the development of biogas infrastructure, and the creation of carbon credit mechanisms to promote sustainability in agricultural practices. Future research should address limitations in geographic variability, indirect effects of biogas adoption, and expand system boundaries to include full supply chain impacts. Incorporating social life-cycle assessment dimensions could further enhance understanding of technology accessibility and community benefits across various socioeconomic contexts.
